# Epigenetic Drifts during Long-Term Intestinal Organoid Culture

**DOI:** 10.3390/cells10071718

**Published:** 2021-07-07

**Authors:** Torsten Thalheim, Susann Siebert, Marianne Quaas, Maria Herberg, Michal R. Schweiger, Gabriela Aust, Joerg Galle

**Affiliations:** 1 Interdisciplinary Center for Bioinformatics (IZBI), Leipzig University, 04107 Leipzig, Germany; herberg@izbi.uni-leipzig.de (M.H.); galle@izbi.uni-leipzig.de (J.G.); 2 Laboratory for Translational Epigenetics and Tumor Genetics, University Hospital Cologne, 50931 Cologne, Germany; susann.siebert@uk-koeln.de (S.S.); mschweig@uni-koeln.de (M.R.S.); 3 Center for Molecular Medicine Cologne (CMMC), 50931 Cologne, Germany; 4 Research Laboratories, Department of Surgery, Leipzig University, 04103 Leipzig, Germany; Marianne.Quaas@medizin.uni-leipzig.de (M.Q.); Gabriela.Aust@medizin.uni-leipzig.de (G.A.)

**Keywords:** age-related drifts, mouse small intestine, MMR-deficient mice, histone modification, H3K4me3, H3K27me3, ChIP-seq, RNA-seq

## Abstract

Organoids retain the morphological and molecular patterns of their tissue of origin, are self-organizing, relatively simple to handle and accessible to genetic engineering. Thus, they represent an optimal tool for studying the mechanisms of tissue maintenance and aging. Long-term expansion under standard growth conditions, however, is accompanied by changes in the growth pattern and kinetics. As a potential explanation of these alterations, epigenetic drifts in organoid culture have been suggested. Here, we studied histone tri-methylation at lysine 4 (H3K4me3) and 27 (H3K27me3) and transcriptome profiles of intestinal organoids derived from mismatch repair (MMR)-deficient and control mice and cultured for 3 and 20 weeks and compared them with data on their tissue of origin. We found that, besides the expected changes in short-term culture, the organoids showed profound changes in their epigenomes also during the long-term culture. The most prominent were epigenetic gene activation by H3K4me3 recruitment to previously unmodified genes and by H3K27me3 loss from originally bivalent genes. We showed that a long-term culture is linked to broad transcriptional changes that indicate an ongoing maturation and metabolic adaptation process. This process was disturbed in MMR-deficient mice, resulting in endoplasmic reticulum (ER) stress and Wnt activation. Our results can be explained in terms of a mathematical model assuming that epigenetic changes during a long-term culture involve DNA demethylation that ceases if the metabolic adaptation is disturbed.

## 1. Introduction

A long-term intestinal organoid culture enables the massive expansion of intestinal stem cells (ISCs) [1,2]. Accordingly, it offers a broad range of medical applications [3] and the chance to overcome the difficulties, as well as the ethical and legal concerns, associated with the use of human fetal stem cells. However, massive in vitro expansion might come with an increased frequency of cell transformation, as indicated by changed organoid growth properties [4]. These growth changes suggest progressive activation of the Wnt pathway. They cannot be considered age-related, because aging of the intestine is associated with reduced Wnt activity [5,6]. In a long-term tumor-derived organoid culture, genetic profiles are preserved [7]. Thus, growth changes in long-term organoid cultures might relate to epigenetic drifts, e.g., drifts of histone or DNA methylation. Such changes are reversible in principle. Indeed, adapted culture conditions, e.g., by supplementing aspirin, have been shown to suppress them [4].

Extrinsic Wnt activation is a general feature of intestinal organoid culture. Only after tumor-like transformation do organoids grow independently of Wnt [8]. Organoid culture media containing EGF, noggin and R-spondin (ENR culture) guarantee a tissue-like cell composition of the organoids and can be adapted to prefer either ISC maintenance or specifications [9]. Recently, we provided a computational model of organoid growth to explain cell compositions under altered Wnt activation [10]. The origin of progressive Wnt activation in organoid cultures, however, remains unresolved. Wnt activation in *Braf^V600E^*-activated colon organoids has been linked to CpG island methylator phenotype-like promoter DNA methylation [8].

The promoters of many genes of the Wnt pathway are known to be associated with bivalent modified nucleosomes [11], i.e., nucleosomes that carry trimethylation of lysine 4 and 27 at histone 3 (H3K4me3 and H3K27me3). In a theoretical approach, we demonstrated that these modifications are sensitive to the cell cycle length [12], providing a potential explanation for long-term modification changes during organoid expansion. As an alternative explanation of epigenetic activation, one might consider stress-induced changes in the ISC pool, as observed following irradiation or in mismatch repair (MMR)-deficient *VC^+/?^Msh2^LoxP/LoxP^* (*Msh2^−/−^*) mice [13]. However, the changes described so far are not associated with gene expression differences in general or with Wnt pathway genes in particular [14].

Here, we study the epigenetic changes associated with the long-term organoid culture of macroscopic normal intestinal tissues. In order to capture the potential effects of genomic stress, we compare MMR-deficient (*Msh2^−/−^*) and control (*Msh2^+/+^*) mice. We focus on the changes of the H3K4me3 and H3K27me3 modifications known to be associated with transcriptional changes and, thus, with the potential regulatory input in the gene expression.

## 2. Materials and Methods

### 2.1. Mice

The mouse strains B6.SJL-Tg(Villin-Cre)997Gum/J and B6.Cg-Msh2tm2.1Rak/J were obtained from The Jackson Laboratory (Bar Harbor, MA, USA). By crossing both, the conditional *Msh2* allele was placed under the control of the *Villin-Cre* transgene. Mice were genotyped as described [15]. *VC^+/?^Msh2^LoxP/LoxP^* (*Msh2^−/−^*) and *VC^−/−^Msh2^flox/flox^* (*Msh2^+/+^*) mice were bred under specific pathogen-free conditions.

### 2.2. Organoid Preparation and Culture 

Six centimeters of the jejunum was used to generate intestinal organoids from 8-month-old *Msh2^−/−^* and *Msh2^+/+^* mice [4]. About 50 isolated crypts were seeded into 10-µL Matrigel/well. Organoids were split once/week and cultured with or without 1-mM aspirin (Sigma Aldrich GmbH, Munich, Germany) for a further 18 weeks. After the first and 18th weeks, organoids were stored in liquid nitrogen. These frozen organoids were re-cultured simultaneously in an IntestiCult™ mouse organoid growth medium (Stemcell Technologies GmbH, Cologne, Germany) with or without 1-mM aspirin for a further two weeks and analyzed afterwards. As the culture time exceeded 72 h, close to 100% of the organoids show a branched growth pattern [4]. Finally, we compared organoids from *Msh2^−/−^* and *Msh2^+/+^* mice at the following ages: 8 months plus 3 weeks of culture, 8 months plus 20 weeks of culture and 8 months plus 20 weeks of culture with aspirin.

### 2.3. Chromatin Immunoprecipitation (ChIP)

Chromatin was prepared up to 50-mg jejunum using SDS shearing buffer and the truChlP Tissue Chromatin Shearing Kit (Covaris, Brighton, UK) according to the manufacturer’s instructions. ChIPs were run on the IP-Star compact system using the Auto iDeal ChIP-seq kit for histones (Diagenode, Seraing, Belgium) according to the manufacturer’s direct method for ChIP preparation.

### 2.4. Quantitative PCR and Illumina Sequencing 

As a quality control for ChIP precipitates, a quantitative real-time PCR analysis was performed in 384-well format in a 10-µL volume using GoTaq qPCR Master Mix (Promega, Mannheim, Germany) and run on a LightCycler 480 (Roche Diagnostics, Mannheim, Germany). The primers used for qPCR are shown in Table S1. The oligonucleotides for *Gapdh* and the “background” were taken from references [16,17], respectively. For all the other primer pairs, publicly available ChIP-seq data were uploaded in UCSC, and the genomic regions showing an enrichment for the respective histone marks were selected for the primer design. The relative enrichment was calculated using the % input method. Library preparations were performed following the instructions of the TrueSeq LT PCR free Kit (Illumina, San Diego, CA, USA) or the TruSeq Nano DNA Kit (Illumina). Sequencing was performed on the Illumina HiSeq 4000 (Illumina) with 76-bp paired-end sequencings.

### 2.5. ChIP-Seq Data Preprocessing and Peak Calling 

The sequenced reads underwent a quality trimming using cutadapt [18] to ensure a minimum read length of 40 bases and a Phred quality score above 20. The quality reports were generated and analyzed using Fastqc [19]. The high-quality reads were mapped to the mouse reference genome NCBI37/mm9 using the software tool segemehl 0.2.0 [20] and its default parameters, except for enabled split/splice detection. Histone modification peaks, i.e., genomic regions in which modifications are enriched, were identified using MACS [21,22].

### 2.6. ChIP-Seq Data Analysis 

After the peak calling process, we generated summarized peak lists for each genotype and treatment. Each of the resulting lists contained the peaks that were either consistently detected in both of the replicates or that had a high reliability (MACS fold enrichment > 5.0). For peaks that were detected in both replicates, the maximum peak breadth and tag density were taken. Subsequently, we identified peaks within the promoter region of the genes (defined as the transcription start site, TSS, +/− 1000 bases) and the gene bodies (defined as the region between the TSS and the last base of the gene). The gene reference list containing 31,592 RefSeq genes was taken from the UCSC Table Browser. To avoid sex-specific artifacts, we excluded genes and peaks of the X- and Y-chromosomes from the analysis. If a peak had a minimum overlap of 5% with a promoter region and/or a gene body, the respective gene-associated histones were considered to carry the modification in a binary present or non-present manner. Thus, each gene received a three-digit histone modification code (0: modification absent and 1: modification present) for each sample. The order of this code was H3K4me3–H3K27me3.

### 2.7. RNA-Seq and Data Preprocessing

The intestinal tissue was homogenized with a Tissue Lyser (20 s at 15 Hz). RNA was prepared following the manufacturer’s recommendations (Qiagen QIA AllPrep kit, Hilden (NRW), Germany), including DNase I digestion. RNA was sequenced on an Illumina HiSeq 4000 sequencer with 75-bp paired-end, strand-specific, random priming. Data preprocessing was performed as described for the ChIP-seq data (see above). Raw counts were normalized to the total number of reads, resulting in one read per million (RPM) values per gene. These RPM values were normalized to the gene lengths (RPKM: reads per kilobase million).

### 2.8. Self-Organizing Map (SOM) Analysis 

The data profiles over all the samples (ChIP-seq: histone codes and RNA-seq: RPKM data) were then clustered into metagene profiles using SOM machine learning, with each metagene serving as a representative of a cluster of combined profiles. We used a grid of 30 × 30 metagenes and applied default parametrization as implemented in oposSOM [23].

### 2.9. Gene Set Enrichment Analysis 

The analysis was performed using the GO enrichment analysis tool PANTHER, Version 12.0 [24]. Data and image analyses were conducted using statistics software R [25].

## 3. Results

### 3.1. Histone Modification Profiles in Organoids 

**Figure 1:**
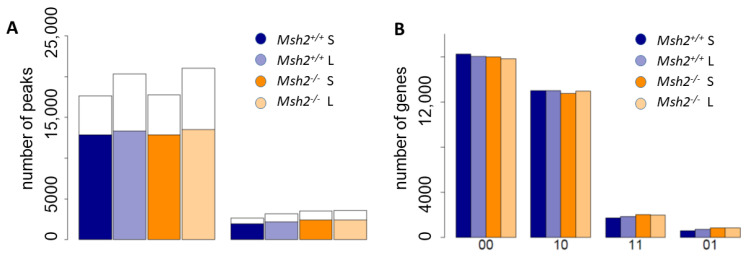
Histone ChIP-seq data. (**A**) Bar plots of the total number of quality peaks (**left**: H3K4me3; **right**: H3K27me3) and the fraction of these peaks associated with the genes (colored parts) for organoids derived from *Msh2^+/+^* and *Msh2^−/−^* mice. Short-term (S) and long-term (L) organoids are compared. (**B**) Distribution of the modification states (H3K4me3 and H3K27me3) among the genes.

To characterize the histone modification profiles of intestinal organoids from 8-month-old mice cultured for 3 (short-term) and 20 (long-term) weeks, we measured the histone marks H3K4me3 and H3K27me3 and the control H3pan by histone ChIP-seq [26]. The organoids were derived from two *Msh2*^+/+^ and *Msh2^−/−^* pairs of mice. Based on these data, we constructed summary lists of the genomic regions in which modified histones were detected for each modification and mouse pair [13]. The modified regions are hereafter referred to as peaks. For further analyses, we selected quality peaks (see: Material and Methods). The number of quality peaks measured was similar in all samples (Figure 1A). The maximum difference we observed between the short- and long-term cultures was an increase by about 16% in the H3K4me3 peaks in the long-term culture. This difference nearly vanished when considering gene-associated peaks only. Each gene obtained a 0/1-doublet code (X Y) depending on whether the associated histones carried a particular modification (1) or not (0). The frequencies of these modification states were also similar among all the samples (Figure 1B).

### 3.2. Epigenetic Profiles of Tissue and Organoids Overlap Considerably 

**Figure 2:**
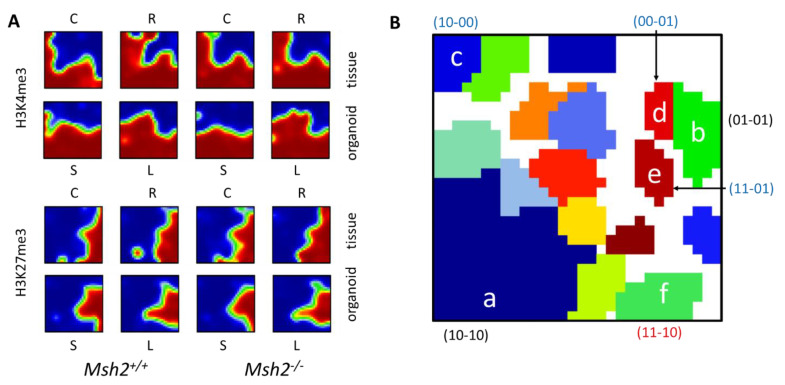
SOM analysis of histone modification states. The analysis integrates the organoid ChIP-seq data presented in this study and published ChIP-seq data on intestinal tissues [13]. (**A**) SOM portraits of all samples (C = control, R = radiated, S = short-term and L = long-term culture). (**B**) Gene clusters with specific regulations. Small letters (a–f) indicate the correlation clusters discussed in the text. The modification states of the cluster-associated genes in the tissue and organoids are indicated in parentheses.

We compared these organoid data with those of irradiated and untreated intestinal tissues of *Msh2*^+/+^ and *Msh2^−/−^* mice [13] to identify culture-specific changes in the organoids. For this purpose, we calculated a self-organizing map (SOM) including all samples of both studies (Figure 2A). Within the SOM, genes with similar regulations in all the samples clustered together. In the following, we discuss selected clusters in detail (clusters **a**–**f**, Figure 2B).

The largest cluster (cluster **a**) comprised 11,627 genes that carried the (10) modification state with high probability in all the samples. The average K4 modification of all genes of the cluster within the individual samples was close to 1. Thus, an extensive overlap existed between the histone profiles of the organoids and tissues. In fact, 96% and 97% of the genes that carried the (10) state in all the organoids and all tissue samples, respectively, were part of cluster **a**. The GO enrichment analysis shows a high enrichment of the cluster **a** genes in the gene sets “protein transport”, “DNA repair” and “cell cycle”. Cluster **b** comprised 502 genes carrying the (01) modification state in all the samples. The average K27 modification of all genes within the individual samples, however, only reached 0.8, indicating a substantial modification variance. These genes wer enriched in the gene sets “ion channel activity” and “G-protein-coupled receptor activity”.

### 3.3. Differences between the Intestinal Tissue and Organoid Culture 

Besides the genes with similar modifications in the tissues and organoids, several subgroups of genes showed one modification profile in all the tissue samples and a different one in all the organoid samples. This property refers to the genes of the clusters **c**–**f** in the SOM (for other profiles, see Figures S1–S3).

**Figure 3:**
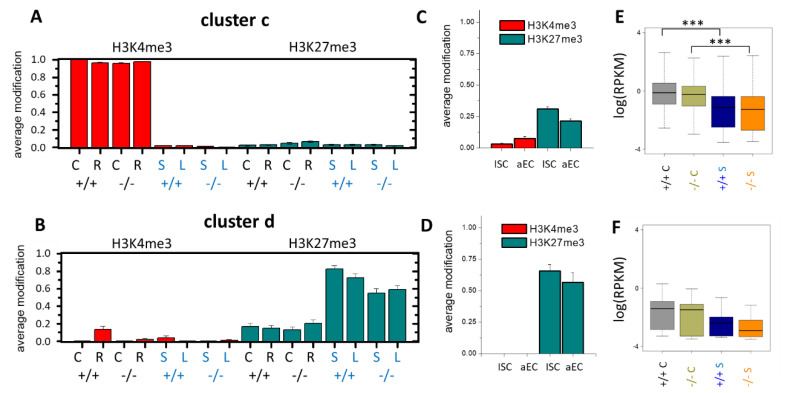
Epigenetic repression of the cluster **c** and **d** genes. Samples: *Msh2^+/+^*: +/+, *Msh2^−/−^*: −/−; black letters: tissue, C = control, R = irradiated, blue letters: organoids, S = short-term and L = long-term culture (error: se). (**A**) Cluster **c** comprises 401 genes in state (10) in all tissue samples that lose H3K4me3 in all organoid samples (00). The fraction of genes associated with the modification is shown. (**B**) Cluster **d** comprises 102 genes in state (00) in all tissue samples that gain H3K27me3 in all organoid samples (01). (**C**,**D**) In isolated ISCs and aECs (Kazakevych et al., 2017), genes of both clusters show the modification state of the organoids (error: se). (**E**,**F**) The changes in the organoids are expected to induce gene repression. A significant change is seen for cluster **c** only. Box plots include expression data of all genes of the clusters in the 3 tissue and 3 short-term organoid samples; *** *p*-value < 0.001 (*t*-test).

Clusters **c** and **d** were associated with genes unmodified in the organoid or tissue samples, respectively. While the genes of cluster **c** lost H3K4me3 in the organoid culture, those of cluster **d** gained H3K27me3 in the organoid culture (Figure 3A,B). To test whether the changes were due to the missing interactions of the epithelial cells with other cell types in the organoids, we compared our results with published data on isolated ISCs and adult enterocytes (aECs) [27]. These ChIP-seq data strongly overlapped with the tissue data applied in the SOM [13]. Isolated ISCs and aECs showed the histone modification profiles seen in the organoids (Figure 3C,D). This observation provided evidence for a nonepithelial origin of the changes in the histone profiles. A prediction based on the modification changes is that the genes of both clusters become transcriptionally downregulated in organoids.

We tested this prediction by performing RNA-seq on the short-term organoid cultures and compared the results with the RNA-seq data on the tissue samples published recently [14]. Most of the genes of cluster **c**, which lost H3K4me3, indeed became repressed in the organoids (Figure 3E). These genes were strongly enriched in the GO gene sets associated with the “immune response”. Such regulations can be expected in organoid cultures. In contrast, the genes of cluster **d**, which gained H3K27me3, showed a weak tendency for repression in the organoids (Figure 3F). These genes were not enriched in any GO gene set.

**Figure 4:**
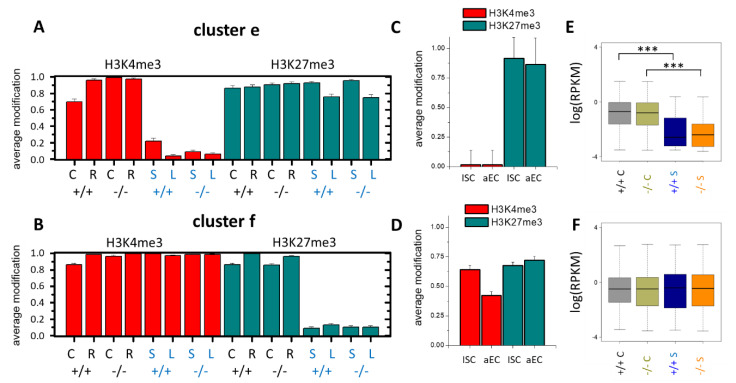
Epigenetic changes in the bivalent genes of clusters **e** and **f**: (**A**) Cluster **e** comprises 342 genes in state (11) in all the tissue samples that lose H3K4me3 in all the organoid samples (01). (**B**) Cluster **f** comprises 147 genes in state (11) in all the tissue samples that lose H3K27me3 in all the organoid samples (10). (**C**,**D**) Modification states in isolated ISCs and aECs based on the published data [27]. (**C**) Close to 100% of cluster **e** genes show the same modification state as in the organoids. (**D**) In contrast, about 60% of cluster **f** genes show the modification state seen in the tissue samples. (**E**,**F**) Box plots of the RNA-seq data of all spot genes in the 3 tissue and 3 short-term organoid samples. (**E**) Cluster **e** genes that lose H3K4me3 are, as expected, transcriptionally repressed. (**F**) The transcription of cluster **f** genes is unchanged; *** *p*-value < 0.001 (*t*-test).

The genes associated with the (11) state, called bivalent genes, are associated with developmental and aging processes. Among the genes carrying the (11) state in the tissue samples and changing their histone modification state in the organoid cultures, one subgroup (cluster **e**, Figure 4A) lost H3K4me3 in the organoid culture, and another subgroup (cluster **f**, Figure 4B) lost H3K27me3. These two subgroups were enriched in the GO gene sets “regulation of ion transport” and “morphogenesis of an epithelium”, respectively.

The genes of cluster **e**, as those of clusters **c** and **d**, showed the same modification profiles in the organoids as in isolated ISCs and aECs, also indicating a nonepithelial origin of these changes (Figure 4C). However, the genes of cluster **f** mainly showed the modification profile of the tissues in these isolated cells, suggesting a culture effect on the epithelium (Figure 4D). As for the genes of cluster **c**, the loss of H3K4me3 in cluster **e** correlated with transcriptional repression (Figure 4E), while a changed H3K27me3 status in cluster **f** was not linked to transcriptional changes on average (Figure 4F). Among the cluster **f** genes were many regulators of the Wnt pathway (*Lgr5, Fosl1, Wnt9a, Wnt10a, Wnt9b, Wnt6* and *Fzd2*). The transcription of *Lgr5, Wnt9b* and *Fzd2*, measured with sufficient reliability by RNA-seq, indicated a profound transcriptional activation (Δlog(T): *Lgr5* 0.98, *Wnt9b* 0.82 and *Fzd2* 0.46), as expected for the organoid culture.

### 3.4. Epigenetic Activation during the Long-Term Organoid Culture 

**Figure 5:**
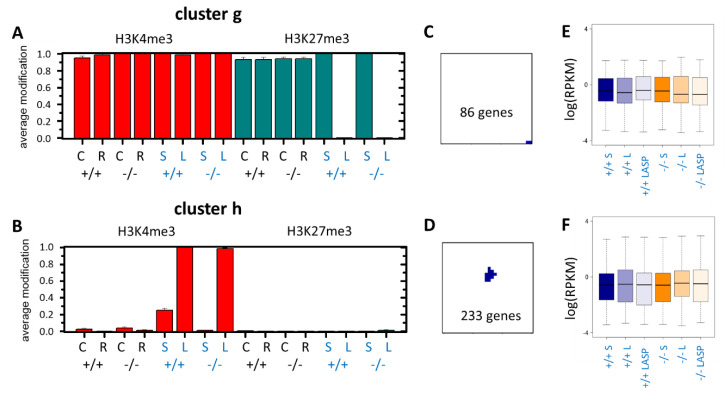
Epigenetic activation during the long-term organoid culture. (**A**) The loss of H3K27me3 at stable H3K4me3 in old organoids occurs in the 86 genes of cluster **g**. (**B**) The gain of H3K4me3 occurs in the 233 genes of cluster **h**. (**C**,**D**) Position of the clusters **g** and **h** in the SOM, respectively. (**E**,**F**) Box plots of the transcription of genes associated with clusters **g** and **h**, respectively. The average transcription of both clusters shows no differences in the short-term (S) culture, long-term culture (L) and long-term cultures supplemented with aspirin (LASP); *Msh2^+/+^*: +/+ and *Msh2^−/−^*: *−/−*.

In addition to the differences between the intestinal tissue and short-term organoid culture, we also observed changes that manifested during the long-term culture only. Two scenarios of epigenetic gene activation were most prominent: (i) the loss of H3K27me3 at genes bivalently modified in the tissue samples (Figure 5A, cluster **g**) and (ii) the gain of H3K4me3 at genes that were unmodified in the tissue samples (Figure 5B, cluster **h**). The genes associated with these regulation types clustered in small spots of the SOM (Figure 5C,D), which were identified by an under-expression spot instead of a correlation cluster analysis [28]. The observed changes occurred in both *Msh2*-deficient and control organoids. Based on the published data [27], we found that (Figure S4): (i) the genes of cluster **g** and **h** did not change their modification states during ISC differentiation into enterocytes. Thus, the observed modification changes could not originate during enterocyte (de-)differentiation. (ii) The average promoter DNA methylation of cluster **g** genes was similar to the other bivalent gene sets observed in normal intestinal tissue, while that of the cluster **h** genes was high, as typically found for unmodified genes that recruit DNA methylation to their promoter during intestinal development [29]. In agreement, the CGs were enriched in cluster **g** but not in cluster **h** genes (Figure S4). The genes themselves were not significantly enriched in the GO sets.

Aspirin decreased the tumor frequency in *Msh2^−/−^* mice [30]. In *Msh2^−/−^* organoids, aspirin reduced the transient cyst-like growth [4]. Based on these observations, we hypothesized that aspirin has the potential to suppress Wnt signaling. Thus, we performed additional RNA-seq analyses of organoids from the long-term culture supplemented with and without aspirin for both *Msh2* genotypes. Comparing the expression in the short- and long-term cultured organoids, we found no differences in the average transcription of cluster **g** and, surprisingly, cluster **h** genes (Figure 5E,F). Notably, the average transcription of the clusters **g** and **h** genes was higher than that of similarly modified gene sets (see: the transcription of cluster **e**, **f** and **d** genes in the tissue, respectively).

### 3.5. Long-Term Organoid Culture-Related Changes in the Transcription 

**Figure 6:**
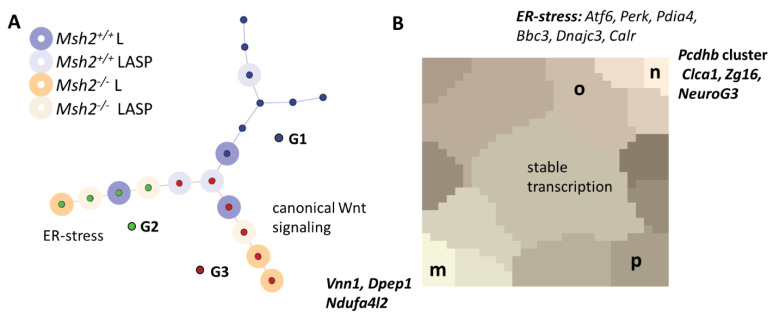
Transcriptional profiles of the organoid samples. (**A**) Correlation spanning tree based on the SOM analysis of RNA-seq data on the short- and long-term organoid cultures (ASP: aspirin-supplemented cultures). Unsupervised clustering identifies 3 groups of samples. Group 1 (G1, blue) comprises all samples from the short-term organoids. Groups 2 (G2, green) and 3 (G3, red) comprise samples of the long-term organoids only. (**B**) K-means cluster of the RNA SOM. Cluster **m** comprises genes that become downregulated during culture, while cluster **n** genes become upregulated. Selected genes are indicated. The clusters **o** and **p** are associated with genes exclusively upregulated in group 2 and group 3, respectively. Their genes are enriched in the GO sets related to “response to endoplasmic reticulum stress” and “canonical Wnt signaling”. Examples are provided for both sets. Genes of other clusters show intermediate regulation.

To characterize the transcriptional profiles of the long-term cultured organoids in more detail, we calculated a “RNA SOM”, including the data on all the organoid samples. The correlation spanning tree based on this SOM showed a clear separation between the short- and long-term organoids (Figure 6A). One branch (group G1) contained all the short-term organoid samples. The long-term organoid samples formed two branches (group G2 and G3). The organoids derived from *Msh2^+/+^* and *Msh2^−/−^* mice did not separate into different branches, although three out of four G2 samples were derived from *Msh2^−/−^* mice. The long-term organoid culture supplemented with aspirin tended to keep more similarities with the short-term culture organoids.

The entire area of the RNA SOM was divided into a set of K-means clusters [28] (Figure 6B). We analyzed these clusters in more detail. The long-term organoid culture induced both gene down- and upregulation, most consistently found in the cluster **m** and **n** genes, respectively. The cluster **m** genes were enriched in the GO set “anchored component of the membrane”. Among them were several genes that are typically expressed by enterocytes, such as *Vnn1* and *Dpep1.* In addition, the hypoxia-inducible gene *Ndufa4l2* [31] that regulates cellular oxygen consumption contributed to this cluster. The cluster **n** genes were enriched in the GO set “homophilic cell adhesion via plasma membrane adhesion molecules”. Among them was the protocadherin cluster *Pcdhb* [32]. In contrast to cluster **m**, cluster **n** comprised genes expressed by secretory lineages such as *Clca1*, *Zg16* and *NeuroG3* [33]. Consistent with our results on the average transcription of cluster **g** and **h** genes, these genes did not accumulate in cluster **n** (Figure S5).

### 3.6. Maturation and Metabolic Adaptation in Long-Term Organoid Culture 

**Figure 7:**
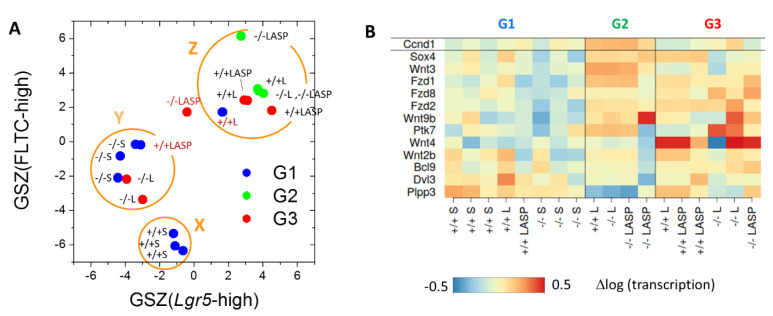
Evidence for maturation and metabolic changes in the long-term organoid culture. (**A**) The gene set Z (GSZ)-scores for Sets I (*Lgr5*-high) and II (FLTC-high) separate the samples from G1-3 into three regulatory states (X, Y and Z circles are to guide the eyes). States X and Y are occupied by G1 samples from *Msh2^+/+^* and *Msh2^−/−^* mice, respectively. Samples that activate sets I and II during the long-term culture occupy state Z. This refers to the G2 samples and *Msh2^+/+^* mouse-derived G3 samples. The G3 samples derived from *Msh2^−/−^* mice show no activation and occupy state Y. Brown labels indicate 3 samples not obeying these rules. (**B**) Heatmap of the transcription of the genes of the canonical Wnt signaling pathway (strongest regulated genes) and its target gene *Ccnd1*. Activation of the Wnt pathway genes is seen in the long-term culture in general. However, only the G2 sample shows uniform target gene activation.

The observed transcriptional changes might be associated with the changing differentiation during the long-term culture. Thus, we analyzed a gene set (set I: *Lgr5*-high, Figure S6) that is characteristic for cells highly expressing the ISC marker *Lgr5* [34]. In the G2 and *Msh2^+/+^* mouse-derived samples of G3, this set was subjected to activation not seen in the G1 or *Msh2^−/−^* mouse-derived samples of G3 (Figure 7A). This activation may refer to maturation of the *Lgr5*-high phenotype and/or an enrichment of *Lgr5*-high cells. The parallel activation of marker genes of secretory cells argued for a maturation (Figure S6). A similar activation as of the set I genes was seen for another, largely disjunct gene set (set II: Fetal Long-Term Culture (FLTC)-high, Figure S6) that characterized the maturation of mouse fetal intestinal tissue-derived organoids during the long-term culture [35]. As gene set II comprised many genes encoding P450 enzymes and transmembrane transporters, this showed, in parallel to maturation, a metabolic adaption in the G2 and *Msh2^+/+^* mouse-derived samples of G3.

While the G2 and *Msh2^+/+^* mouse-derived samples of G3 shared these properties, what separated G2 and G3? The specific activation in G2 referred to the genes associated with cluster **o** of the RNA SOM. In this cluster, genes of the GO set “response to endoplasmic reticulum stress” were enriched (Figure 6B). In contrast, the G3 sample showed the specific activation of the genes associated with cluster **p**. Here, genes of the GO set “canonical Wnt signaling pathway” were enriched. We analyzed the Wnt pathway gene transcriptional profile of G2 and G3 in more detail. While the inspection of these genes unveiled a general trend for activation in the long-term culture (Figure 7B), only the G2 samples showed uniform activation of the Wnt target gene *Ccnd1*, together with the activators of the canonical Wnt pathways *Wnt3* and *Sox4* and a strong repression of the Wnt suppressor *Plpp3* (Figure 7B). In summary, only the G2 samples were characterized by ER stress and Wnt activation.

### 3.7. Epigenetically Activated Genes Respond to Global Transcription 

We found clear differences when comparing the transcription of the epigenetically activated genes sets in G2 and G3, (Figure S5). While a large number of cluster **g** genes were solely activated in G2, the cluster **h** genes were frequently activated in G3 but not in G2. Thus, the expected transcriptional activation was seen in one of the sample groups only. The genes of cluster **f**, in general, showed very diverse transcriptional changes in the long-term culture, including those contributing to the GO set “morphogenesis of an epithelium” (Figure S7). The largest regulated group, however, was activated in G2 only, similar to the cluster **g** genes.

Notably, the Wnt pathway genes of cluster **f** that lost H3K27me3 in the short-term organoid culture only weakly overlapped with the cluster **j** set of the Wnt pathway genes becoming upregulated in G3. The only common Wnt gene was *Lgr5*, which was weakly upregulated during the long-term culture (Figure S7). This implicated that Wnt activation in the short-term culture was different from Wnt adaptation in the long-term culture.

## 4. Discussion

The long-term culture of organoids is associated with different requirements; among them is the adaptation to growth factors and matrices [36] and other environmental changes, such as altered partial oxygen pressure [33]. Moreover, under standard cultures, organoid epithelial cells lack contact to other cell types, including mesenchymal and immune cells [37], and to nonpathological microbes that support ISC maintenance [38]. Here, we studied the epigenetic changes in organoids depending on the culture time and the degree of genetic instability, focusing on the H3K4me3 and H3K27me3 modifications.

In addition to the expected changes in the short-term culture, organoids of both the *Msh2* genotypes showed similar changes of their epigenomes during the long-term culture. These changes were mostly associated with gene activation, i.e., the affected genes showed a gain of H3K4me3 or loss of H3K27me3.

**Figure 8:**
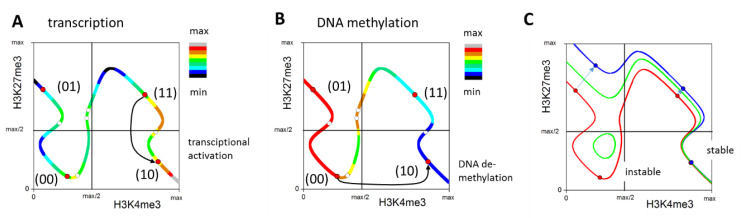
Hypothetical regulation of the cluster **f**, **g** and **h** genes. (**A**,**B**) Phase diagram of the H3K4me3 and H3K27me3 states of a single gene as introduced by reference [12]. The line shows the states that are consistent with histone modification dynamics for high DNA methylation activity. Each state along the line is associated with a specific transcription and DNA methylation (see: color codes in (**A**,**B**), respectively). Only a few states are consistent with the transcriptional regulation of the gene (circles); among them, four are stable fixed points (red circles). The transitions between the fixed points due to fluctuations are common. For the cluster **f** and **g** genes, gene activation by the transcription factors results in a preference for the H3K4me3 state (**A**). The unmodified state is globally stable. Accordingly, in order to leave it, cluster **h** genes require DNA demethylation, rendering the state unstable (**B**). (**C**) Stable fixed points change for the decreasing DNA methylation activity (red: high; green: middle; blue: low activity).

Recently, we described the gain of H3K4me3 to H3K27me3-modified promoters as a stress-related regulation [14]. Nevertheless, H3K4me3 recruitment to unmodified promoters was seen under the same settings, although with lower frequency [13]. Thus, the regulation might represent a similar stress response that occurs during long-term cultures only. Similar changes, however, are seen for genes of an ISC signature during maturation of the fetal intestine [27]. A phase diagram of the H3K4me3 and H3K27me3 modification states as introduced by reference [12] provides an explanation for the underlying regulation (Figure 8A,B). For strong methylation activity, four stable modification states exist: (11), (10), (00) and (01). In the data presented, cluster **h** genes were originally unmodified and showed strong promoter DNA methylation, characteristic for state (00). This state is globally stable and, if attained, cannot be left. This becomes possible following DNA demethylation, as demonstrated for genes of the mentioned ISC signature [27]. Reduced DNA methylation activity renders state (00) instable and can force a switch to state (10) (Figure 8B,C). For highly expressed, unmodified genes, DNA methylation is thereby reduced considerably, while transcription remains largely stable. Although being present in general, such a regulation seems to be more effective in G3, where cluster **h** genes become transcriptionally activated compared to the G2 samples.

While DNA demethylation activates the ISC signature, the gain of H3K27me3 is the major epigenetic process that silences developmental genes [27]. In accordance with these findings, the loss of H3K27me3 from gene promoters and subsequent gene activation has been described as a frequent event in tumorigenesis [11]. In short-term organoid cultures, it is a frequent event as well. Here, it is part of the regulation that enabled ISC expansion by Wnt activation (*Lgr5* activation). In long-term organoid cultures, it continues, somewhat surprisingly, at highly transcribed genes. These genes are atypical H3K27me3 targets, as this modification is preferentially associated with gene silencing [39].

There are several options to induce H3K27me3 loss, including DNA methylation changes, transcriptional gene activation and accelerated proliferation activity [12]. In short-term organoid cultures, it is most likely an effect of the transcriptional activation of genes by growth factors (switch from state (11) to state (10); Figure 8A), e.g., activation of *Lgr5* by R-spondin. We expect a loss of H3K27me3 to progress during a long-term culture whenever it is not impeded by maturation. This is most probable for genes originally highly expressed, in agreement with our observations and under conditions that suppress maturation. Our results suggest that the latter occurs in G2.

The long-term organoid culture activated a transcriptional profile characteristic for intestinal maturation and metabolic adaptation to culture conditions. These changes were seen in all samples from the *Msh2^+/+^* mice. They were partly suppressed in samples from the *Msh2^−/−^* mice. A potential explanation relates to the findings in a colonic monolayer culture [33]. Here, a prolonged culture induced similar changes from a regenerative, fetal-like transcriptional profile towards a mature one. These changes were reversed under hypoxic conditions. In the intestinal organoid culture, hypoxic conditions were induced by cellular oxygen consumption [40]. The intestinal culture from wild-type mice adapted to the hypoxic conditions and increased oxygenation in higher organoid passages [41]. We hypothesize that cells from aged *Msh2^−/−^* mice are less capable of culture adaptation. In this case, the hypoxic conditions remained and induced ER stress, as seen in the G2 samples. If this stress was high, it reversed the ongoing maturation, and the regenerative, fetal-like state of the short-term culture stabilized. This was in agreement with the observations in the colonic monolayer culture [33], where induced ER stress indeed reversed maturation. Moreover, a stabilized fetal-like state explained the increased cyst-like organoid growth particular in aged *Msh2^−/−^* mouse-derived organoid cultures [4].

There are several findings supporting this hypothesis. *Ndufa4l2*, which was transcribed in the short-term culture, can suppress oxidative phosphorylation [31]. Thus, its repression in the long-term culture likely increased the oxygen consumption and exacerbated the hypoxia. This explains why ER stress was observed in the G2 but not in the G1 samples. *Vnn1* is known to control oxidative stress regulation. Actually, *Vnn1* knockout mice have a much higher resistance to oxidative stress than wild-type mice [42]. Thus, the downregulation of *Vnn1* in the long-term culture likely represented a growth advantage under the suggested scenario. The long-term culture activated components of the canonical Wnt-signaling pathway. However, pathway activity (in terms of Ccnd1 transcription) was increased in the G2 samples only. This can be explained by our hypothesis as well, as hypoxia can enforce Wnt activity [43]. Finally, hypoxia is known to abrogate Tet activity [44]. Thus, DNA demethylation, required to activate cluster **h** genes, was suppressed in G2.

Our hypothesis has the following implications. The long-term epigenetic changes are a part of, and not the origin of, the observed maturation process. Thereby, gene activation by a gain of H3K4me3 can be considered as a consequence of DNA demethylation activity, known to be a driving mechanism during ISC maturation [27]. This activation was hindered in G2, where hypoxic conditions suppressed DNA demethylation. Under these conditions, gene activation by a loss of H3K27me3 was facilitated.

The molecular profiles in the short-term organoid culture, compared to their tissues of origin, have been described in detail [45]. Here, we focused on the epigenetic histone H3 trimethylation changes associated with the long-term organoid culture and their consequences in gene transcription, focusing on the RNA-seq data of the gene sets with defined epigenetic regulation. Changes in the gene transcription not associated with these epigenetic gene sets reflected, among other functions, the immediate adaptation to culture conditions. These conditions vary in their supplements and matrices and may also impact the long-term effects. Therefore, the collagen matrix per se induces a stronger *Lgr5* expression compared to Matrigel [46]. An ENR culture supplemented with CHIR99021 and valproic acid showed an even higher *Lgr5* expression in the short-term culture [47]. Notably, this enrichment was lost in the long-term culture.

The in vitro expansion of stem cells for therapeutic use came into focus more than 20 years ago. However, the risks associated with their rigorous expansion to achieve sufficient cell numbers have been addressed only rarely. The activation of the regenerative potential in organoid cultures is associated with broad transcriptional and epigenetic changes. Our study suggests that long-term cultures are accompanied by maturation and adaptation processes that involve both regulatory layers as well. Prolonged genomic stress can suppress these processes.

From our organoid studies, we can conclude that MMR deficiency disturbs the capability of intestinal cells to control their metabolism. This potentially explains why profound transcriptional changes are seen in the macroscopic normal tissue of Lynch syndrome patients [48]. Whether such deregulations are associated with the onset of tumor formation requires further studies.

## Data Availability

The mouse intestinal RNA-seq [14] and ChIP-seq data [13] can be obtained in the Gene Expression Omnibus (GEO) repository (https://www.ncbi.nlm.nih.gov/geo, accessed on 30 Jun 2021) under the super series accession number GSE146520. The organoid ChIP-seq data were deposited in the GEO repository under accession number GSE179237. The RPKM data generated from RNA-seq and the gene cluster **a**–**h** can be obtained in the Leipzig Health Atlas (https://www.health-atlas.de, accessed on 5 July 2021) under the ID 87NG1KD2AT-1.

## Supplementary Materials

The following are available online at https://www.mdpi.com/article/10.3390/cells10071718/s1, Figure S1: Recruitment of H3K4me3 to H3K27me3 targets. Figure S2: Recruitment of H3K4me3 to unmodified genes. Figure S3: Long-term culture-induced recruitment of H3K27me3 to H3K4me3 targets. Figure S4: Properties of gene sets that become epigenetically activated during long-term organoid culture. Figure S5: Genes epigenetically activated in long-term organoid culture show diverse transcriptional behavior. Figure S6: Details on the maturation and adaptation process in long-term organoid culture. Figure S7: Genes epigenetically activated in short-term organoid culture show diverse transcriptional behaviors. Table S1: The primers used for qPCR.
