# Supplementary material for: Epigenetic Drifts during Long-Term Intestinal Organoid Culture
Source: Cells. 2021 Jul 7;10(7):1718. doi: 10.3390/cells10071718 (PMC8305684; doi:10.3390/cells10071718)
Supplement: Supplementary file 1 [file cells-10-01718-s001.zip › cells-1285067-supplementary/cells-1285067-supplementary.pdf]

Supplement 1: Organoid culture induces the 'genomic stress' profile.

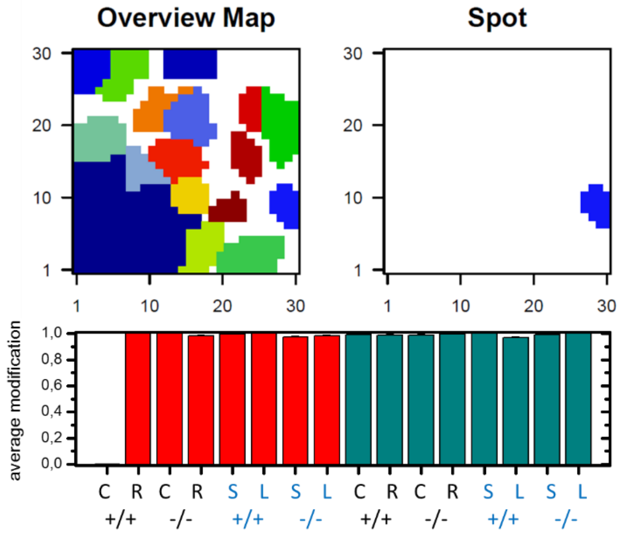

Figure S1: Recruitment of H3K4me3 to H3K27me3 targets. The recruitment was previously described in intestinal tissue under genomic stress [13]. Here, the same recruitment is seen in 254 genes of all organoid samples (>80% of the set seen in stressed tissue alone).

Hypothesis: This suggests that a similar epigenetic stress response is seen under genomic and culture-induced stress.

Supplement 2: Similar gene activation in long-term organoids and irradiated tissue

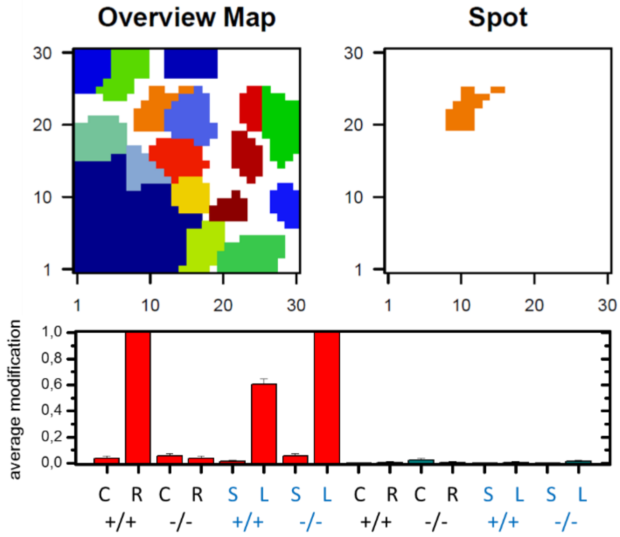

Figure S2. Recruitment of H3K4me3 to unmodified genes. While cluster **h** genes recruit H3K4me3 to previously unmodified genes exclusively in long-term organoid culture, another set of 126 genes recruits this modification to unmodified genes in long-term organoid culture and, in addition, in irradiated tissue of *Msh2*$^{+/+}$ mice.

Hypothesis: This gives rise to the idea that recruitment of this activating mark represents a stress response also in cluster **h** genes.

Supplement 3: Long-term culture induces bivalency

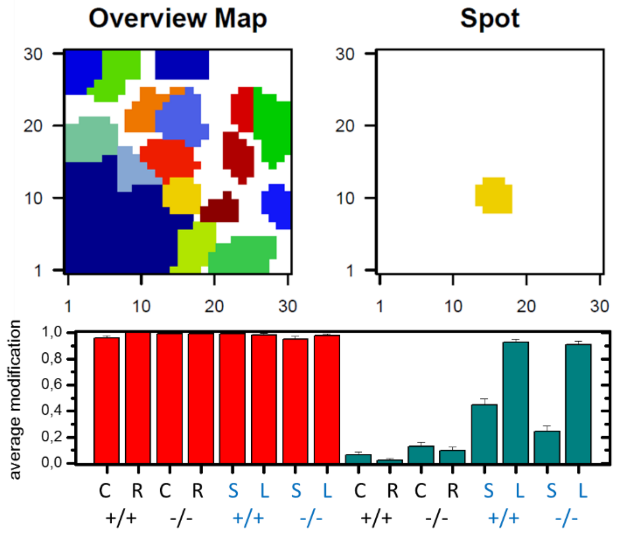

Figure S3: Long-term culture-induced recruitment of H3K27me3 to H3K4me3 targets. Although bivalency is lost at many genes in either short-term (cluster **f**) or long-term culture (cluster **g**), there are 121 genes that acquire this kind of modification state during long-term culture.

Hypothesis: Ongoing maturation of the epithelium is accompanied by progressive epigenetic silencing of developmental genes by H3K27me3.

Supplement 4: Gene sets epigenetically activated during long-term organoid culture

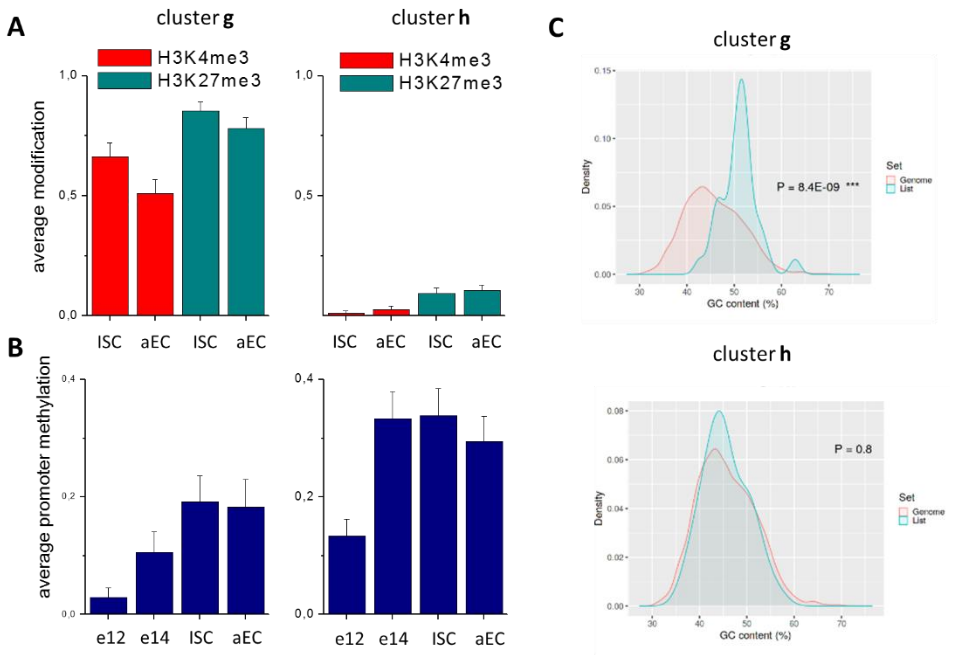

Figure S4: Properties of gene sets that become epigenetically activated during long-term organoid culture. Re-analysis of published data on isolated cells from mice at various ages [27]. A) Histone modification profiles. Clusters **g** and **h** genes conserve their profile during ISC differentiation into adult enterocytes (aECS). B) Promoter DNA methylation. Shown is the average fraction of genes with a DNA methylation peak in their promoter. Cluster **g** genes recruit DNA methylation during development, reaching a value typical for bivalent genes. Cluster **h** genes already reach a high methylation stage at embryonic day e14, typical for aEC. C) Distribution of the GC content of cluster **g** and **h** genes compared to all genes of the mouse genome. Calculation was performed using ShinyGO v0.65 [49].

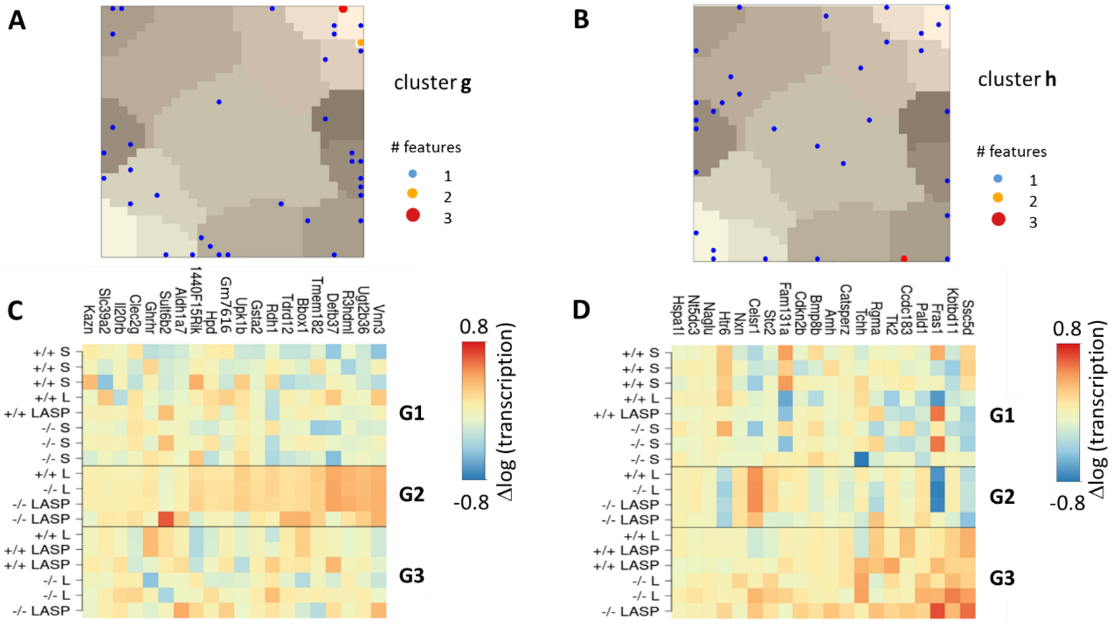

Figure S5. Genes epigenetically activated in long-term organoid culture show diverse transcriptional behavior. A, B) Gene distribution in the SOM. Genes of cluster **g** (A) and cluster **h** (B) do not accumulate in cluster **n**. Gene positions are indicated by colored dots. C, D) Heatmaps of gene transcription. Cluster **g** (C) and cluster **h** genes (D) are preferentially activated in G2 and G3, respectively. Shown are the 20 most strongly regulated genes, ranked according to their transcription in these groups.

Supplement 5: Transcriptional changes of selected gene sets during long-term organoid culture

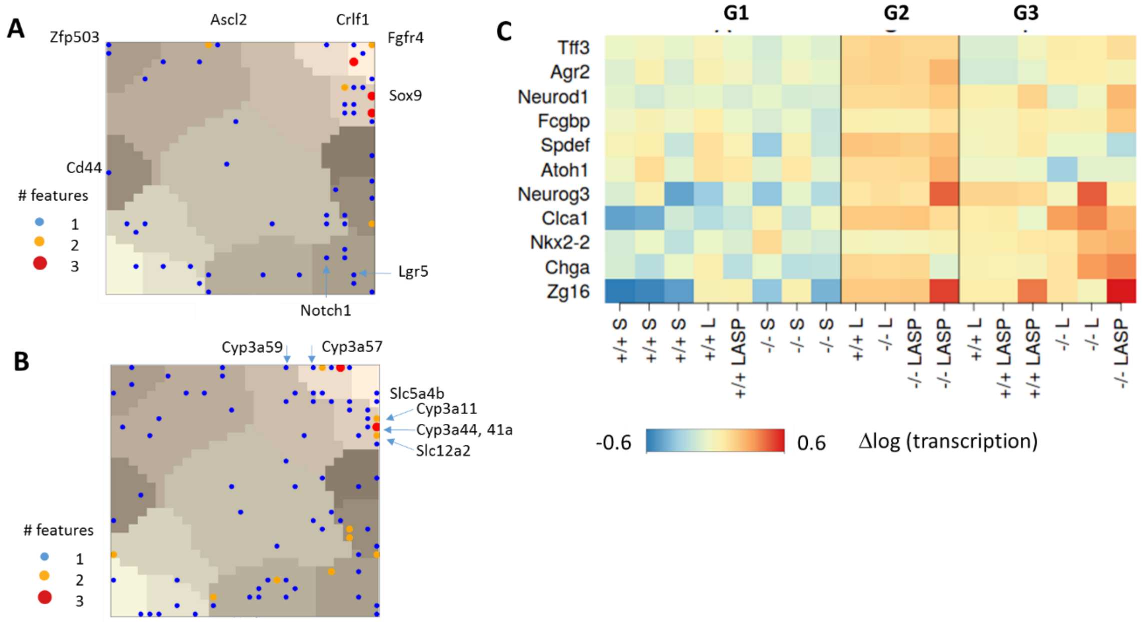

Figure S6: Details on the maturation and adaptation process in long-term organoid culture. A, B) SOM distribution of selected gene sets upregulated in long-term culture. Individual genes are represented by colored dots. Selected genes are labelled. A) Marker set I of *Lgr5*-high expressing cells (**Lgr5**-high). B) Marker set II of genes activated during long-term culture of fetal tissue-derived organoids (FLTC-high). The gene sets are largely disjunct. They overlap in 7 genes only, among them is *Lgr5*. C) Heatmap of the transcription of secretory marker genes that become activated in long-term organoid culture and colonic monolayer culture [33].

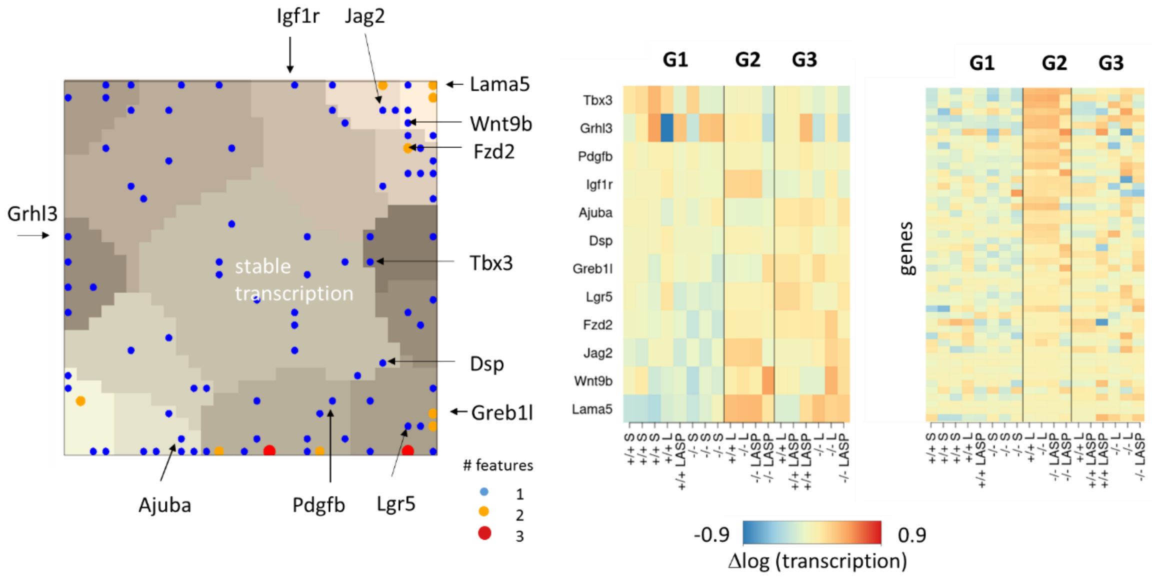

Figure S7: Genes epigenetically activated in short-term organoid culture show diverse transcriptional behaviors. A) Genes that become epigenetically activated in short-term culture by loss of H3K27me3 spread throughout the RNA-SOM (position indicated by colored dots). Genes of the clusters **f** that contribute to the GO set 'morphogenesis of an epithelium' are indicated. B) Heatmap of the transcription of cluster **f** genes contributing to the GO set. C) Heatmap of cluster **f** genes that are activated in G2. They are ranked according to their transcription in this group. Shown are rank 1-50.

Additional references

[49] Ge SX, Jung D, Yao R. ShinyGO: a graphical gene-set enrichment tool for animals and plants. Bioinformatics. 2020; 36(8):2628-2629.
